# Upconversion Nanoparticles Encapsulated with Molecularly Imprinted Amphiphilic Copolymer as a Fluorescent Probe for Specific Biorecognition

**DOI:** 10.3390/polym13203522

**Published:** 2021-10-13

**Authors:** Hsiu-Wen Chien, Chien-Hsin Yang, Yan-Tai Shih, Tzong-Liu Wang

**Affiliations:** 1 Department of Chemical and Materials Engineering, National Kaohsiung University of Science and Technology, Kaohsiung 807, Taiwan; 2 Department of Chemical and Materials Engineering, National University of Kaohsiung, Kaohsiung 811, Taiwan; yangch@nuk.edu.tw (C.-H.Y.); yan.tai.shih@gmail.com (Y.-T.S.)

**Keywords:** upconversion nanoparticles, molecularly imprinted polymers, amphiphilic random copolymer, fluorescence probes

## Abstract

A fluorescent probe for specific biorecognition was prepared by a facile method in which amphiphilic random copolymers were encapsulated with hydrophobic upconversion nanoparticles (UCNPs). This method quickly converted the hydrophobic UCNPs to hydrophilic UNCPs. Moreover, the self-folding ability of the amphiphilic copolymers allowed the formation of molecular imprinting polymers with template-shaped cavities. LiYF_4_:Yb^3+^/Tm^3+^@LiYF_4_:Yb^3+^ UCNP with up-conversion emission in the visible light region was prepared; this step was followed by the synthesis of an amphiphilic random copolymer, poly(methacrylate acid-co-octadecene) (poly(MAA-co-OD)). Combining the UCNPs and poly(MAA-co-OD) with the templates afforded a micelle-like structure. After removing the templates, UCNPs encapsulated with the molecularly imprinted polymer (MIP) (UCNPs@MIP) were obtained. The adsorption capacities of UCNPs@MIP bound with albumin and hemoglobin, respectively, were compared. The results showed that albumin was more easily bound to UCNPs@MIP than to hemoglobin because of the effect of protein conformation. The feasibility of using UCNPs@MIP as a fluorescent probe was also studied. The results showed that the fluorescence was quenched when hemoglobin was adsorbed on UCNPs@MIP; however, this was not observed for albumin. This fluorescence quenching is attributed to Förster resonance energy transfer (FRET) and overlap of the absorption spectrum of hemoglobin with the fluorescence spectrum of UCNPs@MIP. To our knowledge, the encapsulation approach for fabricating the UCNPs@MIP nanocomposite, which was further used as a fluorescent probe, might be the first report on specific biorecognition.

## 1. Introduction

Upconversion nanoparticles (UCNPs) are trivalent lanthanide (Ln^3+^)-doped nanoparticles, which can up-convert two or more lower-energy photons into one high-energy photon [1,2,3,4]. Because Ln^3+^ undergoes f-f transitions within the 4f shell, when Ln^3+^ is embedded in an insulating host lattice, the energies of the excited states will generate a series of states with many closely spaced energy levels, which creates an excited state with a longer lifetime and a sharper optical line shape [2]. Therefore, UCNPs are a type of photostable nanocrystal with a high signal-to-noise ratio, and can be used for photodynamic therapy, light-induced drug delivery, (targeted) cell imaging, and immunoassay sensors [3,4].

The most common strategies for synthesizing small, monodisperse, and bright UCNPs are coprecipitation, thermal decomposition, and solvothermal syntheses [1,5]. Among these strategies, using oleic acid to cap the UCNP is the most common approach. For example, synthesizing hexagonal-phase NaYF_4_ nanoparticles doped with Ln^3+^ involves heating rare earth chlorides in a mixture of octadecene and oleic acid [6,7]. First, oleate salts are generated as in situ precursors. Subsequently, ammonium fluoride and sodium hydroxide are added, and the temperature of the reaction is increased to 300 °C. These processes generate highly monodisperse and oleate-capped UCNPs, which are hydrophobic and can only be dispersed in nonpolar solvents [8].

Nanoparticles for biomedical applications should be water-soluble; to meet this criterion, surficial modifications are usually required [8]. For example, ligand exchange is a well-known method for binding hydrophilic heads to the surface of nanoparticles [9]. Other methods that do not use ligand exchange for nanoparticle solubilization include the formation of a stable silica shell [10] or the use of amphiphilic surfactants or block copolymers to form a bilayer, which occurs via hydrophobic interactions between oleic acid and oleate ions [11,12]. However, if nanoparticles are to be further used as biomarkers, the surfaces of these nanoparticles also need to have distinctive functional groups. Generally, nanoparticles are functionalized after being transferred into water. The common method involves a carbodiimide reaction, which allows amine-containing biomolecules to couple with COOH groups on the surface of the nanoparticles [13,14]. However, 1-ethyl-3-(3-dimethylaminopropyl) carbodiimide hydrochloride (EDC), which is required in this method, is expensive and has a low conversion rate. These shortcomings have therefore stimulated the exploration of other methods of functionalization.

Recently, molecular imprinting for biological recognition has attracted significant attention [15,16,17]. This approach uses the ‘lock and key’ mechanism; therefore, the molecule can identify a cavity with a complementary shape to achieve specific binding. Molecularly imprinted polymers (MIPs) are synthesized by the in-situ polymerization of functional monomers and crosslinkers in the presence of target templates [16]. The monomer can interact with the template through covalent or non-covalent bonds. After copolymerization, chemical decomposition or solvent extraction is used to remove the templates from the polymer network. The resulting cavities retain the corresponding steric and chemical memories of the templates. Therefore, the target species can selectively rebind to MIPs through specific interactions with these imprinted sites [16]. In recent years, some studies have combined MIP with fluorescent nanoparticles to measure fluorescence. The combined system can detect a lower content of the target analytes when compared to MIP only, and the fluorescent nanoparticles provide a readout signal, which improves the sensitivity [18,19,20]. For example, Yu et al. detected acetamiprid with fabricated NaYF_4_:Yb UCNPs encapsulated in a molecularly imprinted polymer [20]. According to their procedure, the UCNPs required surficial modification via a sol–gel method to generate methacrylate groups on the surface of UCNPs. The typical polymerization of MIPs was then applied to UCNPs [20]; however, the procedure is cumbersome.

Folding amphiphilic polymers to form MIPs have been developed recently [21]. In this method, an amphiphilic random copolymer was first synthesized. Next, the amphiphilic random copolymer was mixed and interacted with the templates. The polymer could self-fold to form micelles and encapsulate the templates inside the polymer. After the templates were removed, an imprinted polymer with specific template cavities was formed [21]. Compared with the traditional in-situ polymerization for MIP preparation, this method is easier to remove templates because of the physical interaction between the polymer and the templates. Although a few literature used amphiphilic random copolymers to modify the surface of UCNPs [22], most of them mainly rely on the functional groups of the copolymers graft to UCNPs via the ligand exchange process. In addition, there was no literature that used the combination of amphiphilic random copolymers and UCNP for preparing MIPs. In this study, we propose a strategy that utilizes an amphiphilic random copolymer to encapsulate hydrophobic UCNPs, which does not interfere with the original ligands and can self-fold into water-soluble imprint polymers within the cavities of the template. A oleate-capped LiYF_4_:Yb^3+^/Tm^3+^@LiYF_4_:Yb^3+^ core/shell UCNP with upconversion emission in the visible-light region and amphiphilic poly(methacrylic acid-co-1-octadecene) (poly(MAA-co-OD)) were first synthesized. MIPs are then formed from the surface of the UCNPs modified with the as-prepared amphiphilic poly(MAA-co-OD). This approach not only confers hydrophilicity to the UCNPs, but also integrates specific templates into the polymer shell. Therefore, after dissolution in water, the UCNPs@MIP could be used as a biosensor without further functionalization. When the prepared UCNPs@MIP rebind with specific target analytes within absorption bands of the visible-light region, the fluorescence is quenched, verifying the feasibility of the prepared UCNPs@MIP as fluorescent probes. To the best of our knowledge, this is the first report of molecularly imprinted UCNPs prepared via the folding of amphiphilic random copolymers. 

## 2. Materials and Methods

### 2.1. Materials

Lithium carbonate (Li_2_CO_3_), yttrium(III) oxide (Y_2_O_3_), ytterbium(III) oxide (Yb_2_O_3_), thulium(III) oxide (Tm_2_O_3_), trifluoroacetic acid (TFA), oleic acid, and 1-octadecene (OD) were purchased from Alfa Aesar (Heysham, Lancashire, United Kingdom). Methacrylic acid (MAA) was purchased from SHOWA. Azobisisobutyronitrile (AIBN) was obtained from Aencore (Surrey Hills, Australia). Albumin (Alb), hemoglobin (Hb), and all other chemicals were purchased from Sigma-Aldrich (Saint Louis, MO, USA).

### 2.2. Synthesis of LiYF_4_: Yb^3+^/Tm^3+^@LiYF_4_:Yb^3+^ Core/Shell UCNPs

The LiYF_4_:Yb^3+^/Tm^3+^ core was synthesized by thermal decomposition of lanthanide and lithium trifluoroacetate precursors in the presence of oleic acid, coordinating ligands, and noncoordinating 1-octadecene molecules [23,24]. Briefly, Li_2_CO_3_ (1.44 mmol), Y_2_O_3_ (0.72 mmol), Yb_2_O_3_ (0.25 mmol), and Tm_2_O_3_ (0.01 mmol) were dissolved in 10 mL of aqueous TFA (50%) and stirred at 90 °C until the solution became transparent. Oleic acid (15 mL) and OD (15 mL) were then added to the solution. The resultant solution was then heated to 120 °C under nitrogen gas to remove water and oxygen until the solution turned light yellow. The solution was then heated to 300 °C at a rate of approximately 30 °C/min, and reacted at 300 °C under vigorous stirring for 1 h. The mixture was cooled to room temperature and precipitated with ethanol. The solid was collected by centrifugation at 8000 rpm for 10 min. The solid was then dispersed in n-hexane and re-precipitated with ethanol. The above steps were repeated twice to obtain oleate-capped LiYF_4_:Yb^3+^/Tm^3+^ core nanoparticles (Yields: 88–90%).

For shell growth, Li_2_CO_3_ (1.54 mmol), Y_2_O_3_ (0.77 mmol), and Yb_2_O_3_ (0.2 mmol) were dissolved in 10 mL of aqueous TFA (50%) at 90 °C until the solution became transparent. Oleic acid (15 mL) and OD (15 mL) were then added to the solution. The resultant solution was heated to 120 °C at a rate of approximately 2 °C/min for 30 min under argon gas. The LiYF_4_:Yb^3+^/Tm^3+^ core nanoparticles were then added to the flask. After the solution turned light yellow, it was heated to 300 °C at a rate of approximately 30 °C/min and allowed to react for 1 h. The mixture was cooled to room temperature and precipitated with ethanol. The solid was collected by centrifugation at 8000 rpm for 10 min. The solid was then dispersed and re-precipitated twice with n-hexane and ethanol to obtain the oleate-capped LiYF_4_:Yb^3+^/Tm^3+^@LiYF_4_:Yb^3+^ core/shell UCNPs (Yields: 88–90%).

### 2.3. Synthesis of Amphiphilic Random Copolymer Poly(MAA-co-OD)

A free radical reaction was used to synthesize amphiphilic poly(MAA-co-OD) with a hydrophilic monomer, MAA, and a hydrophobic monomer, OD. Briefly, MAA (0.1 mol), OD (0.1 mol), and AIBN (0.6 mmol) were added to ethanol (100 mL) under nitrogen protection. The polymerization was initiated by heating the mixture at 65 °C for 12 h under constant stirring. After polymerization, the product was purified via precipitation in diethyl ether and centrifugation. After executing this procedure three times, the product was dried under vacuum. The product was further soluble in chloroform for gel permeation chromatography (GPC, YL9100 GPC System, Young Lin Instrument Co., Ltd., Anyang, South Korea) analysis via a polystyrene (PS) standard calibration yielded *M_n_* = 22,894, and a polydispersity of *M_w_*/*M_n_* = 1.507 (Figure S1).

### 2.4. Preparation of UCNPs@MIP

UCNPs@MIP were prepared *via* the encapsulation of amphiphilic random copolymers. Briefly, the poly(MAA-co-OD) (14 mg/mL), as-synthesized hydrophobic UCNPs (8.4 mg/mL), and templates (6 mg/mL) were separately dissolved or dispersed in methanol. The three solutions were mixed at a volume ratio of 1:1:1, and then sonicated for 2 h. The mixture in methanol (2 mL) was then injected into DI water (12 mL), which triggered the self-assembly of the poly(MAA-co-OD) and UCNPs. After the methanol was evaporated, the obtained mixture was centrifuged at 10,000 rpm for 30 min. The precipitate was collected and re-dispersed three times in water to extract the templates, which were Alb and Hb. At the end of this process, a stable colloidal dispersion in water was obtained. For the control experiment, the same procedure without the templates was applied to prepare the non-imprinted polymer (NIP) UCNPs, and the obtained sample is termed UCNPs@NIP.

### 2.5. Characterization of UCNPs and UCNPs@MIP

Wide-angle X-ray diffractograms (WAXD) were obtained with a Bruker D8 ADVANCE diffractometer (Karlsruhe, Germany), using Cu-Kα radiation with a step size of 0.05° and a scanning speed of 4°/min. A JEOL JEM1230 transmission electron microscope (Tokyo, Japan) was used to obtain transmission electron microscopy (TEM) images. A PerkinElmer Lambda 35 UV–vis spectrophotometer (Waltham, MA, USA) was used to perform ultraviolet–visible (UV–vis) spectroscopic analysis. A Hitachi F-7000 fluorescence spectrophotometer (Tokyo, Japan) was used to record the photoluminescence (PL) spectra. A SDL980-LM-5000T laser diode (980 nm, 3 W/cm^2^) from Shanghai Dream Lasers Technology Co., Ltd. (Shanghai, China) was used to obtain the emission spectra of the nanocrystals, after NIR excitation at 980 nm.

### 2.6. Application in Biorecognition

The kinetic adsorption test in this experiment was performed in a tube, where the UCNPs@MIP (50 mg) were mixed with 10 mL of the corresponding template solution (50 mg/mL). The mixed solution was shaken for 0, 15, 30, 45, 60, and 75 min at room temperature. The templates were rebound to the UCNPs@MIP, after which the mixture was centrifuged at 10,000 rpm for 30 min. The supernatant was then collected and analyzed using a UV–vis spectrophotometer. A standard curve was used to calculate the concentration of each sample. The UCNPs@NIP was similarly treated for comparison. The mass balance equation was used to calculate the adsorption capacity (*Q*) [25,26]

$$Q\;\left(\mathrm{mg}/\mathrm{mg}\right)=\frac{(C_{i}-C_{r})V}{m}$$

where *C_i_* (mg/mL) is the initial concentration of the template in the aqueous solution, *C_r_* (mg/mL) is the concentration of the template in the supernatant after adsorption, *m* (mg) is the mass of the adsorbent, and *V* (mL) is the volume of the solution.

For the fluorescence measurements, the templates and UCNPs@MIP were rebound for 45 min, and the mixture was centrifuged at 10,000 rpm for 30 min. The precipitate was collected and re-dispersed in water, and the PL spectra were acquired. The quenching efficiency is expressed as (*F_o_* − *F*)/*F_o_*, where *F_o_* and *F* are the fluorescence intensities at 450 nm, without or with the addition of the template solution, respectively [27,28].

## 3. Results and Discussion

### 3.1. Characterization of the UCNPs

**Figure 1:**
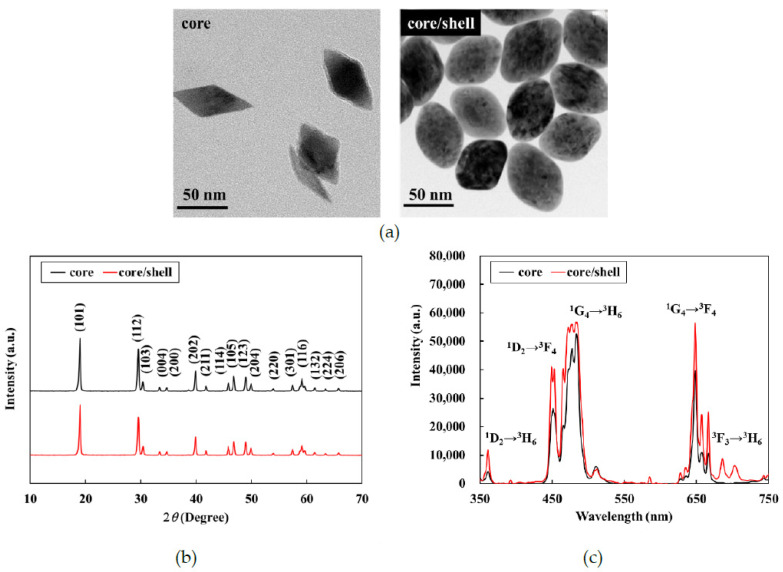
(**a**) TEM images, (**b**) XRD patterns, and (**c**) photoluminescence spectra of LiYF_4_: Yb^3+^/Tm^3+^ core and LiYF_4_: Yb^3+^/Tm^3+^@LiYF_4_:Yb^3+^ core/shell nanoparticles.

The LiYF_4_:Yb^3+^/Tm^3+^@LiYF_4_:Yb^3+^ UCNPs were synthesized *via* thermal decomposition. Tetragonal crystals were readily obtained when the reaction temperature was high [29]; hence, the reaction temperature was set to 300 °C. Based on the TEM images, both the core and core–shell nanoparticles were indeed tetragonal with an octahedral morphology (Figure 1a). The average dimensions of the core nanoparticles, which were calculated from the TEM images, were approximately 75 nm along the long axis and 45 nm along the short axis. After coating the LiYF_4_:Yb^3+^/Tm^3+^ cores with the LiYF_4_:Yb^3+^ shell, the length of the short axis of the core/shell nanoparticles increased to 50 nm. Wide-angle X-ray diffraction (WAXD) was further used to investigate the phase structures of the nanoparticles; peaks were observed at 2θ values of 18°, 29°, 31°, 33°, 34°, 40°, 42°, 46°, 47°, 49°, 50°, 54°, 58°, 59°, 62°, 63°, and 66°, respectively attributed to the (101), (112), (103), (004), (200), (202), (211), (114), (105), (123), (204), (220), (301), (116), (132), (224), and (206) planes of the tetragonal LiYF_4_ crystal (Figure 1b). The relative intensities and positions of all the diffraction peaks in the WAXD pattern were consistent with the Joint Committee on Powder Diffraction Standards (JCPDS) file no. 17e0874, indicating the absence of impurity phases in the synthesized crystals. After coating with the LiYF_4_: Yb^3+^ shell, the XRD patterns of the core–shell nanoparticles were almost the same as those of the core nanoparticles, which indicates that the shell coating did not change the core structure of the crystal. The similarity of the patterns also shows that the shell was likely very thin.

Figure 1c shows that upon excitation at 980 nm, the emission spectra of the LiYF_4_: Yb^3+^/Tm^3+^ core nanoparticles had characteristic emission peaks with electronic transitions at 360 nm (^1^D_2_→^3^H_6_), 450 nm (^1^D_2_→^3^F_4_), 475 nm (^1^G_4_→^3^H_6_), and 650 nm (^1^G_4_→^3^F_4_). This finding confirmed that LiYF_4_:Yb^3+^/Tm^3+^ had upconversion emissions in the region of visible light. After coating with the LiYF_4_: Yb^3+^ shell, the emissions of the core/shell nanoparticles were apparently stronger than the corresponding emissions of the core nanoparticles. During energy transfer, the photoexcited dopants located on or near the surface can be directly deactivated by neighboring quenching centers [30,31]. Moreover, it is plausible that the energy contained in the photoexcited dopants located in the center of the nanophosphors migrated randomly and traveled a long distance on or near the surface of the dopant, or directly to the surficial quenching sites [32,33]. Therefore, the active shell structure plausibly prevented energy quenching, which allowed for high upconversion of the photoluminescence efficiency. Therefore, the core/shell UCNPs could be used in a follow-up study for the encapsulation of MIP.

### 3.2. Hydrophilicity of UCNPs@MIP

**Figure 2:**
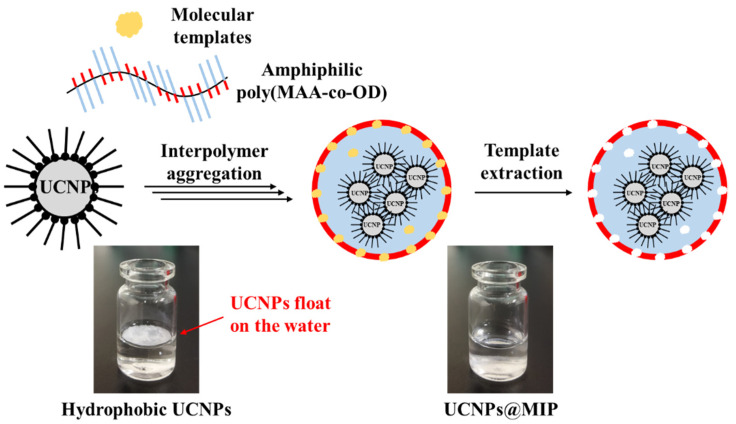
Schematic representation of preparation of UCNPs@MIP.

**Figure 3:**
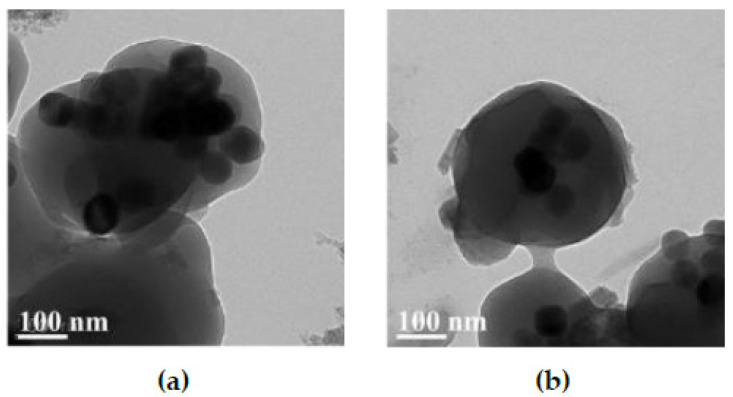
TEM images of UCNPs@MIP. Albumin (**a**) and hemoglobin (**b**) as templates.

Amphiphilic random copolymers of poly(MAA-co-OD) were used to encapsulate the UCNPs, which allowed the poly(MAA-co-OD) to form enclose the UCNPs. Generally, when amphiphilic random copolymers are dissolved in water, the hydrophobic groups self-aggregate via hydrophobic-hydrophobic interactions, while the hydrophilic groups surround the hydrophobic domain; therefore, micelles can be formed and dispersed in water [34]. When the amphiphilic poly(MAA-co-OD) and the hydrophobic UCNPs were mixed, hydrophobic-hydrophobic interactions incorporate the hydrophobic groups in the copolymer with the oleic acid in UCNPs. Consequently, the hydrophilic groups were exposed on the periphery of the hydrophobic aggregates [22]. The water-solubility of the UCNPs, before and after the encapsulation of poly(MAA-co-OD) was visually observed. Figure 2 shows that the hydrophobic UCNPs floated on the water, while the colloidal UCNPs@NIP settled to the bottom of the vial (Figure S2), which indicates that the obtained UCNPs@NIP were water-soluble. Similarly, UCNPs@MIP was also water-soluble, which means that poly(MAA-co-OD) dominated the conversion of hydrophobic UCNPs into hydrophilic, while templates were not a factor to affect the character. TEM was further used to examine the morphology of the prepared UCNPs@MIP (Figure 3). Regardless of whether Ab or Hb was used as a template to prepare the UCNPs@MIP, both types of UCNPs@MIPs were generally composed of several UCNPs, which were aggregated in a polymeric matrix to form quasi-microspheres. The average size of the spheres was approximately 300–500 nm. The results show that the poly(MAA-co-OD) could indeed convert the hydrophobic UCNPs into hydrophilic UCNPs that were capable of forming a micelle-like structure.

Traditional in situ polymerization, which was used to prepare the MIP, involves mixing monomers, crosslinkers, and templates, and then polymerizing and removing the templates to form imprinted polymers with specific template cavities. Recently, folding amphiphilic polymers to form MIPs has attracted attention [21]. In this method, an amphiphilic random copolymer was first synthesized. As the templates interacted with the amphiphilic random copolymer, the polymer self-folded to form micelles and the template structure was imprinted into the polymer. After the templates were removed, an imprinted polymer with specific template cavities was formed [21]. This strategy for folding amphiphilic random copolymers is advantageous. For example, the interaction between the template and the polymer occurs via physical interaction; therefore, the template is easier to remove. This method can use either a hydrophilic or hydrophobic template. Because of the water insolubility of the hydrophobic template, the hydrophobic template becomes completely entrapped inside the micelles. Consequently, the loading capacity of the template and the binding capacity of the MIPs increase [21]. Although there are only a few studies, the superiority of the encapsulation method has been demonstrated. In the present study, the hydrophobic UCNPs were encapsulated with an amphiphilic poly(MAA-co-OD). This approach not only ensured that hydrophilicity was conferred to the UCNPs, but also enabled the integration of specific templates into the polymer shell, which facilitated the formation of MIPs on the surface of the UCNPs. The feasibility of the prepared UCNPs@MIP for molecular recognition is evaluated in the subsequent sections.

### 3.3. Equilibrium Binding of UCNPs@MIP and UCNPs@NIP

**Figure 4:**
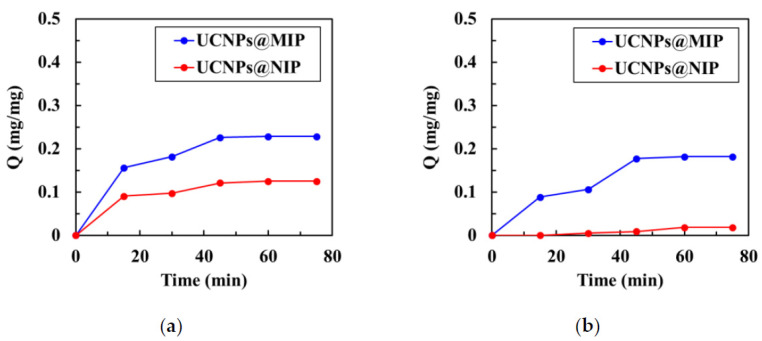
Kinetics of (**a**) albumin and (**b**) hemoglobin adsorption in UCNPs@MIP and UCNPs@NIP.

UV–vis spectroscopy was used to analyze the kinetics of binding of the template molecules with the UCNPs@MIP and UCNPs@NIP. The binding kinetics were used to confirm that the UCNPs@MIPs had the ability to recognize corresponding template molecules. During the recognition of Alb, the first 15 min was the zone of linear increase, and the next 40 min was the saturation zone, regardless of the adsorption curve (UCNPs@MIP or UCNPs@NIP) (Figure 4a). The adsorption capacity of the UCNPs@MIP was much larger than that of the UCNPs@NIPs. This result is noteworthy because it indicates that the UCNPs@MIP had the cavities of Alb, which promoted mass transfer and enhanced rebinding of the template molecules. During the recognition of Hb, the adsorption capacity of UCNPs@NIP increased with the adsorption time (Figure 4b). However, the adsorption capacity was consistently very low; even when the time increased to 45 min, the adsorption capacity was only 0.012 mg/mg. In contrast, the adsorption curve of the UCNPs@MIP showed a linear increase within 40 min, whereas after 40 min, there was a tendency towards saturated adsorption. After 40 min, the adsorption capacity of the UCNPs@MIP was approximately 20 times that of the UCNPs@NIP. These results provide excellent evidence that the imprinting of the UCNPs@MIPs was efficient.

The adsorption capacity of the UCNPs@MIP for Alb was about 0.23 mg/mg at 45 min, compared to 0.18 mg/mg for Hb. This notable result shows that the adsorption capacity of the UCNPs@MIP for Alb was slightly better than that for Hb. In contrast, the adsorption capacity of the UCNPs@NIP for Hb was much smaller than that of Alb. In general, the efficiency of imprinting depended strongly on factors such as the strength of the interactions between the template and the polymeric matrix [26], or the shape of the template. Generally, the hydrophobic residues of most proteins are sequestered in the core of the native structure, while the polar residues are present on the surface [35]. In the structure of Hb, its ‘arm’ is a negatively charged propionate group, which faces the surface of the protein, and the hydrophobic part is buried in the hydrophobic amino acid of the protein. Comparatively, Alb is negatively charged when it is ionized in water at pH 7.4 [36]. Because this experiment used negatively charged MAA as the hydrophilic end of MIP, and Alb and Hb are also negatively charged, the polymer and protein may undergo repulsive interactions, which rationalizes the very low adsorption of Hb on the UCNPs@NIP.

Alb has nominal dimensions of 7.5 × 6.5 × 4.0 nm^3^ and a molecular mass of 66.4 kDa, while Hb has nominal dimensions of 6.0 × 5.0 × 5.0 nm^3^ and a molecular mass of 64 kDa [37]. Although both Alb and Hb are globular proteins [37], Alb is not really spherical, but has a “V” shape [38]. An early study evaluating the adsorption of Alb on silica spheres showed when the V-shaped Alb approached the silica spheres in a direction parallel to the surface of the sphere, it was strongly rejected. However, because albumin has a very small curvature at its V tip, when the V-shaped Alb approached the silica sphere by its tip, it likely penetrated the repulsion field, and was easily adsorbed onto the silica sphere [38]. It is proposed herein that the V-shaped Alb penetrated the repulsive field of UCNPs@MIP or UCNPs@NIP by its tip, which allowed the Alb to enter the polymer matrix. Therefore, Alb adsorption on the UCNPs@NIP was detected. The same rationale likely explains why UCNPs@MIP had a greater adsorption capacity for Alb than for Hb.

### 3.4. Effect of Template Molecules on Fluorescence Quenching

**Figure 5:**
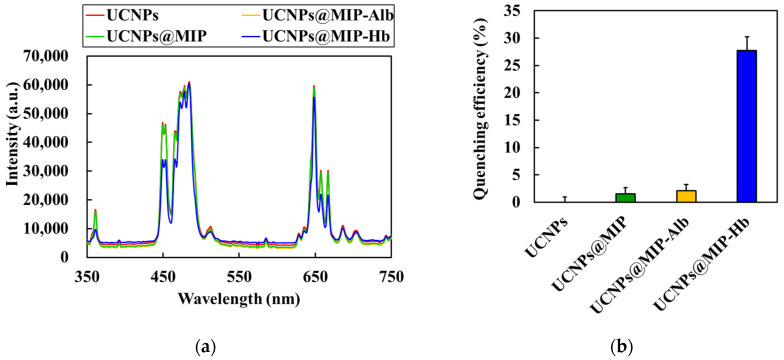
(**a**) Photoluminescence spectra and (**b**) efficiency of fluorescence reduction for the UCNPs, UCNPs@MIP, and UCNPs@MIP bound to albumin and hemoglobin. The quenching efficiency is expressed as (*F_o_* − *F*)/*F_o_*, where *F_o_* and *F* are the fluorescence intensities at 450 nm.

**Figure 6:**
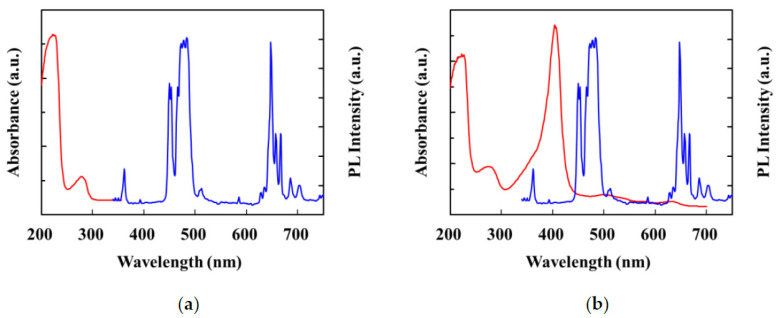
Spectral overlap between the UV–vis absorption spectrum of templates (red line) and the fluorescence spectra of the UCNPs@MIP (blue line). Albumin (**a**) and hemoglobin (**b**) as templates.

The PL spectra of the UCNPs, UCNPs@MIP, and UCNPs@MIP bound to Alb and Hb are shown in Figure 5a. As aforementioned, the emission bands of the as-prepared UCNPs occurred at 360, 450, 475, and 650 nm. The results show that the fluorescence intensity of the encapsulated MIP was similar to that of the original UCNPs, which indicates that the encapsulated poly(MAA-co-OD) did not significantly affect the luminescence of the UCNPs. Furthermore, the fluorescence intensity of the UCNPs@MIP bound to Alb did not change. However, the fluorescence intensity at 360 and 450 nm was evidently reduced when Hb was bound to UCNPs@MIP. The fluorescence quenching may be attributed to Förster resonance energy transfer (FRET) [39,40]. According to Förster’s non-radiative energy transfer theory, energy transfer occurs under the following conditions: (i) the donor can produce fluorescence; (ii) the fluorescence emission spectrum of the donor and the ultraviolet absorption spectrum of the acceptor overlap; (iii) the distance between the donor and the acceptor is <8 nm [41]. As shown in Figure 6b, Hb had six absorption peaks at 220, 280, 340, 400, 550, and 570 nm. The absorption band at 220 nm resulted from the absorption of light between the peptide bonds and amino acid residues. In the UV range of 250–300 nm, light was absorbed by the delocalized electrons of the aromatic side chains. In the wavelength range of 300–700 nm, the absorption of light was associated with the excitation of the porphyrin structure. [42]. Because the absorption spectrum of Hb overlapped with the fluorescence emission spectrum of the UCNPs@MIP, the UCNP emission was quenched by Hb. In contrast, there was no spectral overlap between the absorption spectra of Alb and the emission spectrum of UCNPs@MIP (Figure 6a). Upon binding of the UCNPs to Alb and Hb, the efficiency of fluorescence reduction was approximately 2.1% and 30%, respectively (Figure 5b). The data indicate that the prepared UCNPs@MIP has great potential as a fluorescent probe for detecting biomolecules.

## 4. Conclusions

A facile method for encapsulating hydrophobic UCNPs with amphiphilic random copolymers was proposed. This method could convert the hydrophobic UCNPs to hydrophilic UCNPs, which self-folded to form imprinted polymers with template cavities. The UCNPs with up-conversion emission in the visible-light region of 360 nm and 450–500 nm were first synthesized; this step was followed by the synthesis of an amphiphilic random copolymer of poly(MAA-co-OD). When the UCNPs and poly(MAA-co-OD) were mixed with the templates, a micelle-like structure was formed. After removal of the templates, UCNPs@MIP with a particle size of approximately 500 nm was obtained. The UCNPs@MIP adsorbed more of the template than UCNPs@NIP because the former had template cavities. In addition, UCNPs@MIP had a higher capacity for Alb adsorption than for Hb adsorption because the V-shaped Alb was able to penetrate the repelling field, and was absorbed. Comparatively, because the absorption spectrum of Hb overlapped with the fluorescence spectrum of the UCNPs@MIP, the fluorescence was quenched at wavelengths of 360 nm and 450 nm when Hb was bonded to UCNPs@MIP. The data indicate that the prepared UCNPs@MIP could be used as a fluorescent probe.

## Supplementary Materials

The following are available online at https://www.mdpi.com/article/10.3390/polym13203522/s1, Figure S1: Gel permeation chromatography (GPC) analyses on the synthesized poly(MAA-co-OD). Figure S2: Schematic representation of preparation of UCNPs@NIP.
