# Supplementary material for: Upconversion Nanoparticles Encapsulated with Molecularly Imprinted Amphiphilic Copolymer as a Fluorescent Probe for Specific Biorecognition
Source: Polymers (Basel). 2021 Oct 13;13(20):3522. doi: 10.3390/polym13203522 (PMC8539580; doi:10.3390/polym13203522)
Supplement: Supplementary file 1 [file polymers-13-03522-s001.zip › polymers-1408224-supplementary.pdf]

Upconversion nanoparticles encapsulated with molecularly imprinted amphiphilic copolymer as a fluorescent probe for specific biorecognition

Hsiu-Wen Chien*, Chien-Hsin Yang, Yan-Tai Shih, Tzong-Liu Wang*

[1]Department of Chemical and Materials Engineering, National Kaohsiung University of Science and Technology, Kaohsiung 807, Taiwan

[2]Department of Chemical and Materials Engineering, National University of Kaohsiung, Kaohsiung 811, Taiwan

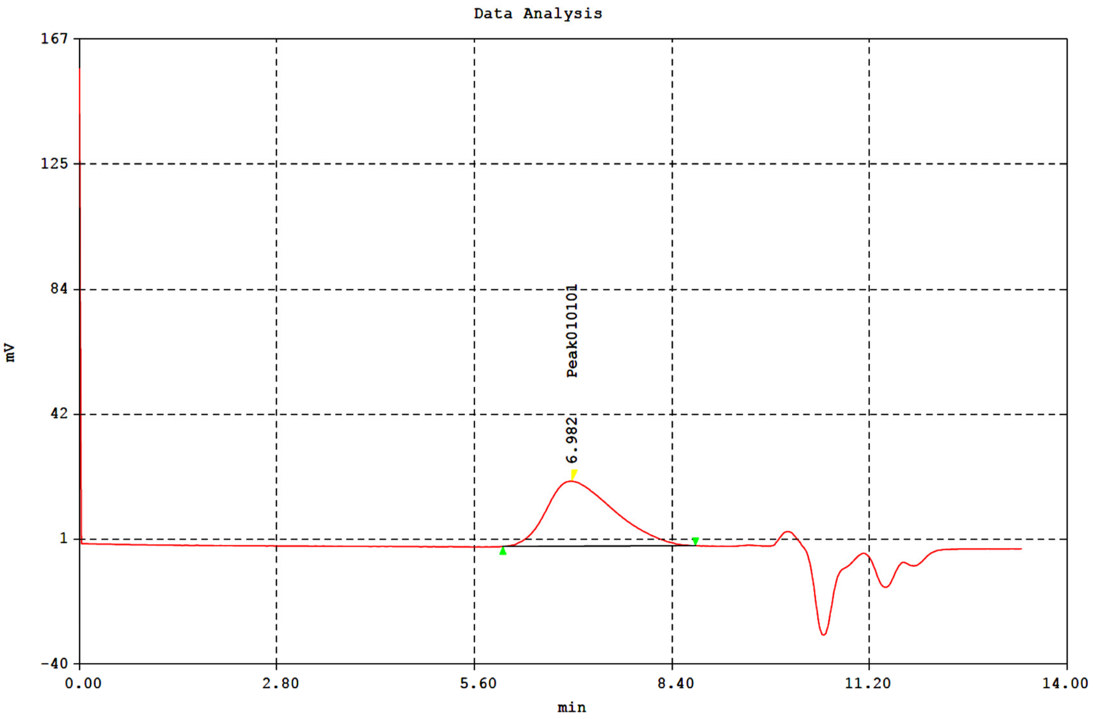

Quantitation Information

Calibration CurvPS-TLW20160809 Adjustment MethNONE

Analysis Result

#	Peak	Mn	Mp	Mw	Mz	Mz+1	Mv	PD	IV
1	Peak010101	22894	39627	34518	45117	54125	N/A	1.50770	N/A

Figure S1 Gel permeation chromatography (GPC) analyses on the synthesized poly(MAA-co-OD)

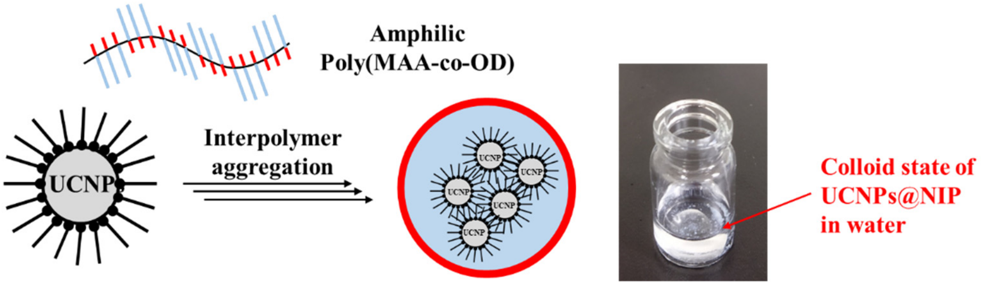

Figure S2 Schematic representation of preparation of UCNPs@NIP.
